# Abdominal Obesity Indices as Predictors of Psychiatric Morbidity in a Large-Scale Taiwanese Cohort

**DOI:** 10.3390/nu18010013

**Published:** 2025-12-19

**Authors:** Jia-In Lee, Jiun-Hung Geng, Yi-Ching Lo, Szu-Chia Chen, Yi-Ya Fang, Cheng-Sheng Chen

**Affiliations:** 1 Graduate Institute of Medicine, College of Medicine, Kaohsiung Medical University, Kaohsiung 80708, Taiwanyichlo@kmu.edu.tw (Y.-C.L.); 2 Department of Psychiatry, Kaohsiung Medical University Gangshan Hospital, Kaohsiung Medical University, Kaohsiung 807378, Taiwan; 3 Department of Psychiatry, Kaohsiung Medical University Hospital, Kaohsiung Medical University, Kaohsiung 807, Taiwan; 4 Department of Urology, Kaohsiung Municipal Siaogang Hospital, Kaohsiung 812, Taiwan; 5 Department of Urology, Kaohsiung Medical University Hospital, Kaohsiung Medical University, Kaohsiung 80756, Taiwan; 6 Graduate Institute of Clinical Medicine, College of Medicine, Kaohsiung Medical University, Kaohsiung 80756, Taiwan; 7 Research Center for Environmental Medicine, Kaohsiung Medical University, Kaohsiung 80708, Taiwan; 8 Graduate Institute of Natural Products, College of Pharmacy, Kaohsiung Medical University, Kaohsiung 80756, Taiwan; 9 Department of Medical Research, Kaohsiung Medical University Hospital, Kaohsiung 80708, Taiwan; 10 Department of Pharmacology, School of Medicine, College of Medicine, Kaohsiung Medical University, Kaohsiung 80708, Taiwan; 11 Department of Internal Medicine, Kaohsiung Municipal Siaogang Hospital, Kaohsiung Medical University, Kaohsiung 80756, Taiwan; 12 Division of Nephrology, Department of Internal Medicine, Kaohsiung Medical University Hospital, Kaohsiung Medical University, Kaohsiung 80708, Taiwan; 13 Faculty of Medicine, College of Medicine, Kaohsiung Medical University, Kaohsiung 80708, Taiwan

**Keywords:** abdominal obesity, psychiatric symptoms, obesity-related indices, depression, anxiety, population-based study

## Abstract

Background/Objectives: Obesity has been linked to a number of diseases, including depression and anxiety. In addition to the commonly used body mass index (BMI) and waist circumference, many obesity-related indices have been proposed. We aimed to investigate the associations between 10 obesity-related indices and psychiatric morbidity in a large cohort of 121,601 Taiwanese participants. Methods: This cross-sectional study analyzed data from 121,601 adults aged 30–70 years enrolled in the Taiwan Biobank between 2012 and 2023. The mean age of the participants was 50 years, and the 10 obesity-related indices were BMI, waist circumference, waist-to-height ratio, waist-to-hip ratio, abdominal volume index, body roundness index, lipid accumulation product, visceral adiposity index, conicity index and triglyceride glucose index. Psychiatric morbidity, defined as the presence of depressive or anxiety symptoms, was identified using self-reported, physician-diagnosed depression, Patient Health Questionnaire 2-item (PHQ-2) score ≥ 3, or Generalized Anxiety Disorder 2-item (GAD-2) score ≥ 3. Multivariable logistic regression models were used to assess associations between each obesity-related index and psychiatric morbidity. Results: Psychiatric morbidity was observed in 5414 (5%), 1375 (3.0%) and 4039 (5%) individuals in the whole cohort, male participants and female participants, respectively. After adjusting for variables, all of the obesity-related indices were significantly associated with psychiatric morbidity, except for BMI in the male subjects. There were no significant interactions between sex and these 10 obesity-related indices. Conclusions: We found significant associations between multiple obesity-related indices and psychiatric morbidity; as these indices are simple and routinely collected, they may help identify individuals at higher psychological risk in population settings. Further research is warranted to clarify underlying mechanisms and their potential utility in screening or prevention.

## 1. Introduction

Obesity is a prominent global public health issue and its prevalence continues to increase. According to the World Health Organization, more than 1 billion people worldwide were classified as individuals with obesity in 2022. Numerous physical diseases including hypertension, cardiovascular diseases, diabetes, musculoskeletal disorders and some cancers have been shown to be strongly associated with, or even caused by, obesity [1]. Obesity is also highly comorbid with depression and anxiety [2,3], and this relationship is thought to be reciprocal because obesity and mood disorders share overlapping behavioral and biological pathways, including chronic inflammation and hypothalamic–pituitary–adrenal (HPA) axis dysregulation [4,5,6]. Because anxiety and depression differ in symptom profiles and biological patterns, their associations with obesity may also vary; this is an important consideration when interpreting heterogeneous effect sizes [2,3,4,5,6].

Anxiety and depression are major global health problems. Recent international estimates show that hundreds of millions of individuals worldwide are affected by these conditions, and similar trends are observed in Taiwan [7,8]. However, the prevalence in Taiwan may be underestimated because some individuals hesitate to report psychological symptoms due to cultural stigma, and emotional distress is often expressed as physical discomfort, such as fatigue, headaches, or gastrointestinal complaints, rather than as mood-related symptoms. These somatic presentations, which are more common in East Asian populations, can delay recognition of anxiety or depression and contribute to underreporting [7]. Mental illness tends to cause profound impairments in social, occupational, and self-care functioning and contributes substantially to the healthcare burden [8]. In population-based studies, PHQ-2, GAD-2, and self-reported physician diagnosis are widely used and validated screening tools, allowing for a pragmatic and consistent definition of psychiatric morbidity [9,10].

In view of the high prevalence and detrimental effects of obesity, anxiety and depression, we were interested in exploring their association. Psychological factors such as emotional eating, body image dissatisfaction, and guilt-related behaviors may further interact with metabolic dysregulation, linking abdominal adiposity to psychiatric vulnerability [11,12]. In addition to the commonly used body mass index (BMI) and waist circumference (WC), many other obesity-related indices have been proposed, including waist-to-height ratio (WHtR), waist-to-hip ratio (WHR), abdominal volume index (AVI), body roundness index (BRI), lipid accumulation product (LAP), visceral adiposity index (VAI), conicity index and triglyceride glucose index (TyG index) [13,14,15]. These indices capture different dimensions of abdominal adiposity, including visceral fat accumulation, metabolic load, and lipid–glucose dysregulation, which may relate differently to mental health outcomes and therefore provide a more comprehensive assessment than BMI or WC alone [13,14,15]. Although many studies have investigated associations between these obesity-related indices with physical diseases. Their associations with anxiety and depression have not been fully investigated in large-scale studies. Thus, in this article, we aimed to explore the association between these 10 obesity-related indices and psychiatric morbidity using a large population-based database in Taiwan.

## 2. Materials and Methods

### 2.1. Data Source and Study Population

The data for this study were obtained from the Taiwan Biobank (TWB), a nationwide population-based research resource established in 2008 to investigate genetic, environmental, and lifestyle determinants of common chronic diseases in Taiwanese adults. TWB recruits community-dwelling volunteers aged 30–70 years who have no history of cancer at enrollment. Participants undergo standardized interviews, physical examinations, and laboratory assessments, and follow-up surveys are conducted regularly.

For the present study, we used data from TWB participants enrolled between 2012 and 2023, during which more than 120,000 individuals had completed baseline assessments. A total of 121,601 participants with complete anthropometric, laboratory, and psychiatric questionnaire data were included in the final analysis.

Inclusion criteria were as follows:

(1) Age 30–70 years at enrollment;
(2) Participation in the Taiwan Biobank baseline survey;
(3) Availability of complete data for obesity-related indices and psychiatric measures.

Exclusion criteria were as follows:

(1) A prior diagnosis of cancer (as per TWB recruitment criteria);
(2) Missing values for any primary exposure or outcome variables.

This study was conducted in accordance with the Declaration of Helsinki and was approved by the Institutional Review Board of Kaohsiung Medical University Hospital (Approval number: KMUHIRB-E(I)-20210058).

### 2.2. Definition and Assessments of the Obesity-Related Indices

During the Taiwan Biobank recruitment, the participants’ anthropometric data and lipid profile were recorded, including weight, standing height, WC and hip circumference. Lipid profile included total cholesterol, triglycerides, high-density lipoprotein and low-density lipoprotein. In this study, we investigated the following 10 obesity-related indices: BMI, WC, WHtR, WHR, AVI, BRI, LAP, VAI, conicity index and TyG index.

WC was measured as the circumference of the midpoint between the iliac crest and the lowest rib. Hip circumference was the largest circumference around the buttocks in a standing position. BMI was weight in kilograms divided by the square of height in meters. WHtR was calculated as WC in centimeters divided by height in centimeters. WHR was calculated as WC divided by hip circumstance. The other indices (AVI, BRI, LAP, VAI, conicity index, and TyG index) were calculated as below based on previous studies.

AVI = 2WC^2^ (cm) + 0.7(WC − Hip Circumference)^2^ (cm)]/1000 [16,17]

BRI = 364.2 − 365.5 [1 − π − 2WC^2^ (m) Height^−2^ (m)]^1/2^ [18]

LAP = (WC [cm] − 65) × (triglyceride concentration [mM]) for men, and (WC [cm] − 58) × (triglyceride concentration [mM]) for women [19]

VAI = (WC [cm]/(39.68 + (1.88 × BMI) × (TG/1.03) × (1.31/high-density lipoprotein) for men, (WC [cm]/(36.58 + (1.89 × BMI) × (TG/0.81) × (1.52/high-density lipoprotein) for women [20]

Conicity Index = 0.109^−1^ WC (m) [Weight (kg)/Height (m)]^−1/2^ [21]

TyG index = Ln[fasting triglycerides (mg/dL) × fasting glucose (mg/dL)/2] [22]

These indices were selected because they capture complementary dimensions of abdominal adiposity beyond traditional measures [11,12,13]. BMI and WC reflect general body size, whereas WHR, WHtR, and the conicity index indicate central or visceral fat distribution. AVI and BRI describe abdominal geometry, while LAP and VAI combine anthropometric and lipid components to represent visceral fat-related metabolic activity. The TyG index reflects insulin resistance and lipid-glucose dysregulation (Supplementary Table S1) [13,14,15].

Because the purpose of this study was to evaluate the continuous relationship between each obesity-related index and psychiatric morbidity, all ten indices were analyzed as continuous variables rather than categorized using clinical cutoffs. This approach is consistent with prior epidemiological studies examining anthropometric indices and mental health and avoids imposing arbitrary thresholds that may not be clinically validated [23,24]. Accordingly, each result reflects the adjusted odds ratio per unit increase in the corresponding index. The cohort distributions of these indices are shown in Table 1, which provides mean values and variability for all ten measures.

### 2.3. Psychiatric Morbidity

Psychiatric morbidity was defined as meeting any of the following criteria: (1) a self-reported physician diagnosis of depressive disorder, (2) a Patient Health Questionnaire (PHQ-2) score ≥ 3, or (3) a Generalized Anxiety Disorder 2-item (GAD-2) score ≥ 3. Depressive and anxiety symptoms were classified jointly as psychiatric morbidity to provide a pragmatic and consistent measure of overall psychological burden in this population. Because both PHQ-2 and GAD-2 are validated screening instruments, a cutoff of ≥3 has been widely recommended for identifying clinically relevant symptoms in large-scale community settings [9,10]. We also acknowledge that relying on self-reported diagnoses may introduce misclassification, and this limitation was considered when interpreting the findings.

Participants were asked whether they had ever been diagnosed with depressive disorder, and those who responded affirmatively were further queried regarding the timing of the diagnosis and any prescribed medications. To complement the self-report measure, standardized symptom-based screening tools were administered. The PHQ-2 [9] assesses the frequency of depressed mood and anhedonia over the past two weeks, with each item scored from 0 (“not at all”) to 3 (“nearly every day”). Total scores range from 0 to 6, and a score ≥ 3 indicates possible depressive disorder. Similarly, the GAD-2 [10] evaluates the frequency of anxiety and uncontrollable worry using the same 0–3 response scale, and a total score ≥ 3 suggests clinically relevant anxiety symptoms warranting further evaluation. Both instruments were administered by well-trained interviewers following standardized procedures [9,10].

### 2.4. Statistical Analyses

Descriptive statistics were used to characterize the profiles of all participants. Continuous variables were presented as means and standard deviations, while categorical variables were shown as numbers and percentages. The participants were divided into two groups based on sex and the presence or absence of psychiatric morbidity. Group differences were evaluated using independent *t*-tests and chi-square tests. Univariate and multivariate logistic regression analyses were conducted to assess the associations between the obesity-related indices and psychiatric morbidity. To ensure interpretability, multicollinearity among indices and covariates was assessed using variance inflation factors (VIF), all of which were below 5, indicating no concerning multicollinearity [25] (Supplementary Table S2). Model fit was assessed using the Hosmer–Lemeshow goodness-of-fit test, acknowledging that this statistic is highly sensitive to large sample sizes and may yield significant *p*-values despite adequate model performance [26] (Supplementary Table S3). A two-side *p*-value of less than 0.05 was deemed statistically significant. All analyses were carried out using R (version 3.6.2, R Foundation for Statistical Computing, Vienna, Austria) and SPSS (version 20.0, IBM Corp, Armonk, NY, USA).

## 3. Results

### 3.1. Clinical Profile of the Participants

A total of 121,601 participants including 43,699 men (35.9%) and 77,902 women (64.1%) were recruited for the study. The mean age was 50 ± 11 years, and there was no significant difference in marital status between the men and women. The male group consumed significantly more alcohol, had a longer smoking history, and reported slightly higher rates of regular exercise compared with the female group. Regarding physical condition, the male group had higher systolic blood pressure, lower diastolic blood pressure, and more prevalent physical diseases including hypertension, diabetes, dyslipidemia, coronary artery disease, chronic obstructive pulmonary disease, gastroesophageal reflux disease, irritable bowel syndrome, gout and chronic kidney disease (Table 1).

### 3.2. Differences in Clinical Characteristics by Sex and Psychiatric Morbidity

The male and female groups were further stratified into those with and without psychiatry morbidity. In the male group, those with psychiatry morbidity had a longer smoking history than those without psychiatry morbidity, and more of those without psychiatry morbidity were married. In the female group, those without psychiatry morbidity had lower rates of ever smoking and alcohol use and higher educational level than those with psychiatry morbidity. In addition, both the males and females without psychiatry morbidity had lower rates of hypertension, diabetes, dyslipidemia, coronary artery disease, chronic obstructive pulmonary disease, gastroesophageal reflux disease, and irritable bowel syndrome (Table 2). All of the obesity-related indices except BMI were significantly lower in both the males and females without psychiatry morbidity, with no significant sex differences. BMI was only significantly lower in the female group (Table 2).

### 3.3. Univariate and Age-Adjusted Analysis for the Association Between Obesity-Related Indices and Psychiatric Morbidity in the Males and Females

Univariate logistic regression analysis was used to explore associations between the obesity-related indices and psychiatric morbidity. After adjusting for age, all of the indices except BMI were significantly associated with psychiatric morbidity in the male group, with a 1.002- to 23.25-fold increased risk (Table 3). Among them, conicity index (odds ratio [OR] = 23.215; 95% confidence interval [95% CI] [9.998, 53.906]), WHR (OR = 16.146; 95% CI [6.070, 42.952]), and WHtR (OR = 7.821; 95% CI [3.018, 20.267]) demonstrated strong effects. High conicity index was associated with a 23-fold increased risk of psychiatric morbidity in men.

In addition, all of the obesity-related indices were significantly associated with psychiatric morbidity in the female group after age-adjusted analysis, as shown in Table 4. Similarly, WHR (OR = 3.876; 95% CI [2.410, 6.234]), conicity index (OR = 3.335; 95% CI [2.277, 4.884]), and WHtR (OR = 3.205; 95% CI [1.945, 5.280]) had the strongest associations.

### 3.4. Multivariate Analysis for the Association Between the Obesity-Related Indices and Psychiatric Morbidity in the Males and Females

The results of multivariate logistic regression analysis for the association between obesity-related indices and psychiatric morbidity are shown in Table 5. In men, all obesity-related indices except BMI and VAI were positively associated with psychiatric morbidity. Indices that reflect central adiposity, particularly the conicity index, WHR, and WHtR, showed the largest effect sizes, indicating substantially elevated risk among individuals with higher abdominal adiposity. Similar patterns were observed in women, although the magnitude of associations was generally smaller. To aid comparison across indices, we generated a summary table presenting adjusted odds ratios with 95% confidence intervals for men and women (Supplementary Table S4), which highlights the consistently stronger associations for central adiposity measures, especially WHR, WHtR, and the conicity index, compared with general obesity markers such as BMI and WC. Sex-by-index interaction terms were not statistically significant for any index, suggesting that differences in effect sizes between men and women were not statistically meaningful. Model diagnostics indicated no concerning multicollinearity (Supplementary Table S2). Although several female models yielded significant Hosmer–Lemeshow *p*-values, this was expected given the large sample size, and no meaningful model misfit was detected (Supplementary Table S3).

## 4. Discussion

In this study, we examined the associations between ten obesity-related indices and psychiatric morbidity, defined by depressive or anxiety symptoms. Across both sexes, conicity index, WHR, and WHtR showed the strongest associations, whereas BMI demonstrated little or no association after adjustment. These height- and hip-adjusted indices likely capture central adiposity more accurately than general body size measures, which may explain their consistently stronger relationships with psychiatric morbidity. Collectively, our findings highlight that indicators reflecting abdominal adiposity, not overall adiposity, show the clearest associations with psychological symptoms in this population.

It is worth noting that the WC is a common factor in the conicity index, WHR, and WHtR. This may suggest that abdominal obesity was the major factor contributing to the risk of psychiatric morbidity. However, WC was associated with a much lower risk compared to conicity ratio, WHR, and WHtR in all results. Although WC is a straightforward indicator of abdominal obesity, it may underestimate the risk in those with a smaller build. Over the past decades, these three indices, adjusted by hip circumference or height, have gradually been shown to be better health indicators to predict numerous comorbidities across different setting and population [15,27,28,29,30,31]. Several studies have also reported stronger associations between central adiposity indices and psychiatric symptoms. For example, WHR and WHtR have been linked to higher depressive and anxiety symptom burdens independent of BMI [32,33,34], and the conicity index has been associated with psychological distress and cardiometabolic dysregulation that may predispose individuals to mood disturbances [35]. Consistent results were reported in a previous systematic review [32], which found that individuals with abdominal obesity had a 1.38-fold increased risk of depression. In addition, another study [33] with a large sample size also found that elevated WHR was associated with an increased prevalence of both anxiety and depression. The authors concluded that abdominal obesity but not general body mass increased the risk of mood disturbance. This point of view was supported by Xu et al. in 2011 [32], who suggested that the relationship between abdominal obesity and depression was stronger than that between general obesity and depression. The potential mechanisms for the relationship between abdominal obesity and depression/anxiety have been broadly discussed [3,36]. One proposed mechanism is related to hypothalamic–pituitary–adrenal axis (HPA-axis) dysregulation [37,38,39] with chronic inflammation and hypercortisolemia or insulin resistance activating the HPA axis [40]. Another potential mechanism includes the antidepressant effects of endocrine hormone leptin [41], which is also considered to play a vital role in HPA regulation [42,43].

We also found that the ORs of these three indices were much higher in the male group than in the female group. These sex differences have been explored before, however the results have been inconsistent. A study of the UK Biobank [44] found that adiposity was associated with probable major depression, and that the association was stronger in women than in men. Another study by Li et al. [45] showed that depressed women had greater BMI and total body fat, while depressed men had greater visceral fat mass. Importantly, indices that capture central adiposity (such as WHR and WHtR) may reflect sex-specific fat distribution patterns more accurately than BMI, which may partly explain the magnitude differences observed in our study [33,34]. The underlying mechanism for these gender differences remains unclear. In addition, another study [46] in Japan found that Japanese men have more visceral adipose tissue than Caucasian men at the same level of WC. Therefore, ethnic differences may also require consideration when discussing sex differences. Furthermore, estrogen plays important roles in fat distribution [47], levels of inflammation [48] and lifestyle factors [49]. Age may also serve as a mediator in the sex differences, and should also be considered. Although sex-related patterns were observed, they should be interpreted with caution. Lifestyle, hormonal, and cultural factors not captured in our dataset may contribute to these differences, suggesting that the observed patterns likely reflect multiple interacting influences rather than biological determinism.

There are several strengths to this study. First, the sample size was large, with more than 120,000 individuals. Second, to the best of our knowledge, this study is the first to investigate the relationship between obesity and psychiatric morbidity using 10 obesity-related indices. Third, all of the obesity-related indices were evaluated by experienced staff with well-validated instruments, which minimizes self-reported errors. Finally, all of the participants were recruited in Taiwan, and our results may reflect the current clinical conditions in the country and help to develop good public health strategies. Nevertheless, there are also some limitations to our study. First, due to the cross-sectional study design, we could not define causality between the obesity-related indices and psychiatry morbidity. Further, more extensive longitudinal studies are warranted to clarify this issue. In addition, reverse causation remains possible, as psychiatric symptoms may influence weight patterns and fat distribution in ways that cannot be disentangled within a cross-sectional framework. Second, the presence of psychiatry morbidity was based on participants’ self-reports without a formal diagnosis by a psychiatrist. Although the validity of self-reported questionnaires has been demonstrated before, more complete evaluations might be considered in future studies. In addition, despite adjustment for multiple covariates, residual confounding from unmeasured factors, such as dietary habits, sleep quality, psychological stress, socioeconomic status, and medication use, may have influenced the observed associations. Third, the participants were ethnically homogeneous, which may limit the generalizability of our findings to populations outside Taiwan, particularly because fat distribution patterns vary across ethnic groups. It is relevant that mental health stigma and culturally influenced reporting behaviors may affect the accuracy of self-reported psychiatric symptoms, further limiting external applicability. Fourth, we were unable to incorporate basal metabolic rate (BMR) because Taiwan Biobank lacks the required measurements (resting metabolic rate or calorimetry-based estimates). Future cohorts with metabolic chamber or accelerometer-based energy expenditure assessments would enable a more comprehensive understanding of metabolic dysfunction in psychiatric morbidity. Fifth, some confidence intervals were relatively wide, particularly in sex-stratified analyses. These wider intervals likely reflect greater variability and reduced statistical precision in certain subgroups due to smaller sample sizes or underlying heterogeneity. Therefore, the magnitude of the associations in these strata should be interpreted with caution. Sixth, nutritional factors may influence both adiposity and psychiatric risk. Prior studies show that dietary patterns affect visceral fat, inflammation, and depressive or anxiety symptoms [50,51,52,53]. Because detailed dietary data were unavailable, we could not assess whether diet mediated or modified these associations. Future studies incorporating nutritional assessments are warranted. Finally, the baseline demographics were different between the male and female groups, which may have introduced bias. We therefore used multivariate analysis to try and mitigate the potential impact.

The future clinical implication of this study is that healthcare providers should consider screening individuals with abdominal obesity for symptoms of depression and anxiety with easily obtained obesity-related indices including conicity index, WHR, and WHtR. By identifying and addressing these comorbid conditions early on, healthcare providers can help improve overall patient outcomes and prevent further health complications. In addition, healthcare providers may consider a multidisciplinary approach to treatment that addresses both the physical and mental health aspects of abdominal obesity, as this may lead to more effective and comprehensive care.

## 5. Conclusions

In this study, we investigated the associations between ten obesity-related indices and psychiatric morbidity. All indices except BMI were associated with increased odds of psychiatric symptoms, with conicity index, WHR, and WHtR showing the strongest relationships, underscoring the relevance of abdominal obesity. Given the cross-sectional design and modest effect sizes, these findings should be interpreted cautiously. Longitudinal studies are needed to clarify causal pathways and to determine whether abdominal adiposity differentially influences anxiety and depressive outcomes. Although these indices may have potential value in risk stratification, further validation is required before they can be applied clinically.

## Figures and Tables

**Table 1:** Profiles of participants classified by sex.

Characteristics	Total (n = 121,601)	Male (n = 43,699)	Female (n = 77,902)	*p* Value
Age, year	50 ± 11	50 ± 11	50 ± 11	0.865
Smoke, ever, n (%)	33,156 (27)	25,081 (57)	8075 (10)	<0.001
Alcohol status, ever, n (%)	10,357 (9)	8172 (19)	2185 (3)	<0.001
Regular exercise, yes, n (%)	49,304 (41)	18,510 (42)	30,794 (40)	<0.001
Married, yes, n (%)	105,059 (86)	37,793 (87)	67,266 (86)	0.502
Education status, ≥College, n (%)	70,475 (58)	29,162 (67)	41,313 (53)	<0.001
SBP (mm Hg)	120 ± 19	126 ± 17	117 ± 19	<0.001
DBP (mm Hg)	74 ± 11	72 ± 11	81 ± 11	<0.001
Hypertension, n (%)	14,887 (12)	7342 (17)	7545 (10)	<0.001
Diabetes, n (%)	6276 (5)	2968 (7)	3308 (4)	<0.001
Dyslipidemia, n (%)	9041 (7)	4059 (9)	4982 (6)	<0.001
CAD, n (%)	1562 (1)	1025 (2)	537 (1)	<0.001
COPD, n (%)	1390 (1)	610 (1)	780 (1)	<0.001
GERD, n (%)	16,666 (14)	5694 (13)	10,972 (14)	<0.001
IBS, n (%)	3026 (3)	1216 (3)	1810 (2)	<0.001
Gout, n (%)	4675 (4)	4239 (10)	436 (1)	<0.001
CKD, n (%)	1951 (2)	1187 (3)	764 (1)	<0.001
Obesity-related indices				
BMI (kg/m^2^)	24 ± 4	25 ± 4	24 ± 4	<0.001
WC (cm)	83 ± 10	88 ± 9	81 ± 10	<0.001
WHtR	0.5 ± 0.06	0.5 ± 0.06	0.5 ± 0.06	<0.001
WHR	0.9 ± 0.06	0.9 ± 0.06	0.8 ± 0.07	<0.001
AVI	14.2 ± 3.5	15.7 ± 3.4	13.4 ± 3.2	<0.001
BRI	3.7 ± 1.2	3.8 ± 1.1	3.7 ± 1.3	<0.001
LAP	32.4 ± 34.7	38.8 ± 42.2	28.8 ± 29.0	<0.001
VAI	1.7 ± 1.9	1.8 ± 2.2	1.7 ± 1.8	<0.001
Conicity index	1.2 ± 0.08	1.2 ± 0.07	1.6 ± 0.09	<0.001
TyG index	8.4 ± 0.6	8.6 ± 0.6	8.3 ± 0.6	<0.001

Continuous variables are presented as mean ± standard deviation; categorical variables as number (percentage). *p* values were obtained using independent *t*-tests for continuous variables and chi-square tests for categorical variables. Abbreviations: SBP, systolic blood pressure; DBP, diastolic blood pressure; CAD, coronary artery disease; COPD, chronic obstructive pulmonary disease; GERD, gastroesophageal reflux disease; IBS, irritable bowel syndrome; CKD, chronic kidney disease; BMI, body mass index; WHtR, waist-to-height ratio; WHR, waist–hip ratio; AVI, abdominal volume index; BRI, body roundness index; LAP, lipid accumulation product; VAI, visceral adiposity index and TyG index, triglyceride glucose index.

**Table 2:** Clinical characteristics of the study participants classified by the presence of different sex and psychiatric morbidity.

	Male			Female		
Characteristics	Psychiatric Morbidity (−)	Psychiatric Morbidity (+)	*p* Value	Psychiatric Morbidity (−)	Psychiatric Morbidity (+)	*p* Value
Age, year	50 ± 11	50 ± 11	0.689	50 ± 11	50 ± 10	0.019
Smoke, ever, n (%)	24,176 (57)	905 (66)	<0.001	7270 (10)	805 (20)	<0.001
Alcohol status, ever, n (%)	7890 (19)	282 (21)	0.084	1989 (3)	196 (5)	<0.001
Regular exercise, yes, n (%)	17,960 (42)	550 (40)	0.076	29,202 (40)	1592 (39)	0.894
Married, yes, n (%)	36,718 (87)	1075 (78)	<0.001	63,805 (86)	3461 (86)	0.212
Education status, ≥College, n (%)	28,271 (67)	891 (65)	0.103	39,366 (53)	1947 (48)	<0.001
SBP (mm Hg)	126 ± 17	125 ± 17	0.013	117 ± 19	117 ± 18	0.041
DBP (mm Hg)	78 ± 11	78 ± 11	0.224	71 ± 11	71 ± 11	0.512
Hypertension, n (%)	7052 (17)	290 (21)	<0.001	7031 (10)	514 (13)	<0.001
Diabetes, n (%)	2834 (7)	134 (10)	<0.001	3045 (4)	263 (7)	<0.001
Dyslipidemia, n (%)	3844 (9)	215 (16)	<0.001	4563 (6)	419 (10)	<0.001
CAD, n (%)	969 (2)	56 (4)	<0.001	473 (1)	64 (2)	<0.001
COPD, n (%)	571 (1)	39 (3)	<0.001	695 (1)	85 (2)	<0.001
GERD, n (%)	5352 (13)	342 (25)	<0.001	9940 (14)	1032 (26)	<0.001
IBS, n (%)	1101 (3)	115 (8)	<0.001	1566 (2)	244 (6)	<0.001
Gout, n (%)	4099 (10)	140 (10)	0.550	413 (1)	23 (1)	0.926
CKD, n (%)	1144 (3)	43 (3)	0.355	707 (1)	57 (1)	0.005
Obesity-related indices						
BMI (kg/m^2^)	24 ± 4	25 ± 4	0.402	24 ± 4	24 ± 4	0.015
WC (cm)	88 ± 9	89 ± 10	<0.001	81 ± 10	82 ± 10	<0.001
WHtR	0.5 ± 0.06	0.5 ± 0.06	<0.001	0.5 ± 0.06	0.5 ± 0.07	<0.001
WHR	0.9 ± 0.06	0.9 ± 0.06	<0.001	0.8 ± 0.07	0.9 ± 0.07	<0.001
AVI	15.7 ± 3.3	16.1 ± 3.7	<0.001	13.4 ± 3.2	13.7 ± 3.5	<0.001
BRI	3.8 ± 1.1	3.9 ± 1.2	<0.001	3.7 ± 1.3	3.8 ± 1.4	<0.001
LAP	38.6 ± 42.1	43.1 ± 44.5	<0.001	28.6 ± 28.8	31.8 ± 31.0	<0.001
VAI	1.8 ± 2.2	2.0 ± 2.0	0.006	1.6 ± 1.8	1.8 ± 1.8	<0.001
Conicity index	1.2 ± 0.07	1.2 ± 0.07	<0.001	1.2 ± 0.09	1.2 ± 0.09	<0.001
TyG index	8.6 ± 0.6	8.7 ± 0.6	<0.001	8.3 ± 0.6	8.4 ± 0.6	<0.001

Continuous variables are presented as mean ± standard deviation; categorical variables as number (percentage). *p* values were obtained using independent *t*-tests for continuous variables and chi-square tests for categorical variables. Abbreviations: SBP, systolic blood pressure; DBP, diastolic blood pressure; CAD, coronary artery disease; COPD, chronic obstructive pulmonary disease; GERD, gastroesophageal reflux disease; IBS, irritable bowel syndrome; CKD, chronic kidney disease; BMI, body mass index; WHtR, waist-to-height ratio; WHR, waist–hip ratio; AVI, abdominal volume index; BRI, body roundness index; LAP, lipid accumulation product; VAI, visceral adiposity index and TyG index, triglyceride glucose index.

**Table 3:** Association of obesity-related indices with psychiatric morbidity using univariate and age-adjusted logistic regression analysis in male participants.

Obesity-Related Indices	Male			Male		
	Crude			Age-Adjusted		
	OR	95% CI	*p*	OR	95% CI	*p*
BMI (kg/m^2^)	1.007	[0.992, 1.022]	0.367	1.007	[0.992, 1.022]	0.383
WC (cm)	1.012	[1.006, 1.017]	<0.001	1.012	[1.006, 1.017]	<0.001
WHtR	7.366	[2.857, 18.992]	<0.001	7.821	[3.018, 20.267]	<0.001
WHR	12.907	[4.994, 33.355]	<0.001	16.146	[6.070, 42.952]	<0.001
AVI	1.033	[1.018, 1.049]	<0.001	1.003	[1.018, 1.049]	<0.001
BRI	1.108	[1.058, 1.160]	<0.001	1.110	[1.060, 1.163]	<0.001
LAP	1.002	[1.001, 1.003]	<0.001	1.002	[1.001, 1.003]	<0.001
VAI	1.021	[1.006, 1.038]	0.008	1.021	[1.006, 1.037]	0.008
Conicity index	18.093	[8.006, 40.889]	<0.001	23.215	[9.998, 53.906]	<0.001
TyG index	1.213	[1.116, 1.317]	<0.001	1.214	[1.117, 1.319]	<0.001

Values expressed as odds ratio (OR) and 95% confidence interval (CI). Abbreviations are the same as in Table 1.

**Table 4:** Association of obesity-related indices with psychiatric morbidity using univariate and age-adjusted logistic regression analysis in female participants.

Obesity-Related Indices	Female			Female		
	Crude			Age-Adjusted		
	OR	95% CI	*p*	OR	95% CI	*p*
BMI (kg/m^2^)	1.011	[1.003, 1.019]	0.009	1.010	[1.002, 1.019]	0.015
WC (cm)	1.009	[1.006, 1.012]	<0.001	1.009	[1.006, 1.012]	<0.001
WHtR	3.430	[2.120, 5.549]	<0.001	3.205	[1.945, 5.280]	<0.001
WHR	3.983	[2.544, 6.237]	<0.001	3.876	[2.410, 6.234]	<0.001
AVI	1.028	[1.019, 1.038]	<0.001	1.027	[1.018, 1.037]	<0.001
BRI	1.065	[1.040, 1.090]	<0.001	1.061	[1.036, 1.087]	<0.001
LAP	1.003	[1.002, 1.004]	<0.001	1.003	[1.002, 1.004]	<0.001
VAI	1.033	[1.020, 1.047]	<0.001	1.032	[1.019, 1.046]	<0.001
Conicity index	3.408	[2.368, 4.905]	<0.001	3.335	[2.277, 4.884]	<0.001
TyG index	1.192	[1.132, 1.256]	<0.001	1.189	[1.126, 1.256]	<0.001

Values expressed as odds ratio (OR) and 95% confidence interval (CI). Abbreviations are the same as in Table 1.

**Table 5:** Association of obesity-related indices with psychiatric morbidity using multivariate logistic regression analysis.

Obesity-Related Indices	Male			Female			
	Multivariate			Multivariate			
	OR	95% CI	*p*	OR	95% CI	*p*	Interaction *p*
BMI (kg/m^2^)	0.997	[0.981, 1.013]	0.709	1.002	[0.993, 1.011]	0.609	0.296
WC (cm)	1.007	[1.001, 1.013]	0.020	1.006	[1.002, 1.009]	0.002	0.626
WHtR	3.515	[1.263, 9.782]	0.016	1.987	[1.158, 3.408]	0.013	0.762
WHR	6.312	[2.211, 18.023]	0.001	2.259	[1.364, 3.739]	0.002	0.745
AVI	1.020	[1.004, 1.037]	0.016	1.017	[1.007, 1.027]	0.001	0.643
BRI	1.065	[1.013, 1.119]	0.013	1.036	[1.009, 1.063]	0.008	0.860
LAP	1.001	[1.000, 1.002]	0.047	1.002	[1.001, 1.003]	0.002	0.140
VAI	1.014	[0.995, 1.033]	0.142	1.017	[1.002, 1.032]	0.027	0.425
Conicity index	10.601	[4.418, 25.437]	<0.001	2.421	[1.633, 3.589]	<0.001	0.172
TyG index	1.134	[1.036, 1.241]	0.006	1.094	[1.031, 1.161]	0.003	0.469

Values expressed as odds ratio (OR) and 95% confidence interval. Abbreviations are the same as in Table 1. Covariates in the multivariate model included age, smoking status, drinking status, exercise habit, married status, educational status, systolic blood pressure, diastolic blood pressure, hypertension, diabetes, dyslipidemia, coronary artery disease, chronic obstructive pulmonary disease, gastroesophageal reflux disease, irritable bowel syndrome, gout and chronic kidney disease.

## Data Availability

The data underlying this study is from the Taiwan Biobank. Due to restrictions placed on the data by the Personal Information Protection Act of Taiwan, the minimal data set cannot be made publicly available. Data may be available upon request to interested researchers. Please send data requests to: Szu-Chia Chen, Division of Nephrology, Department of Internal Medicine, Kaohsiung Medical University Hospital, Kaohsiung Medical University.

## Supplementary Materials

The following supporting information can be downloaded at: https://www.mdpi.com/article/10.3390/nu18010013/s1, Table S1: Conceptual domains reflected by the ten obesity-related indices; Table S2. Variance Inflation Factors (VIFs) for Obesity-Related Indices in Sex-Stratified Logistic Regression Models; Table S3. Hosmer-Lemeshow Goodness-of-Fit Statistics for Sex-Stratified Logistic Regression Models; Table S4. Comparison of adjusted odds ratios (ORs) and 95% confidence intervals for psychiatric morbidity across ten obesity-related indices, by sex.
