# Supplementary material for: Abdominal Obesity Indices as Predictors of Psychiatric Morbidity in a Large-Scale Taiwanese Cohort
Source: Nutrients. 2025 Dec 19;18(1):13. doi: 10.3390/nu18010013 (PMC12787333; doi:10.3390/nu18010013)
Supplement: Supplementary file 1 [file nutrients-18-00013-s001.zip › nutrients-4021720-supplementary.pdf]

Abdominal Obesity Indices as Predictors of Psychiatric Morbidity in a Large-Scale Taiwanese Cohort

Supplementary Table S1. Conceptual domains reflected by the ten obesity-related indices

**Index**	**Primary Domain Reflected**
BMI	General body size
WC	Abdominal girth / general central fat
WHR	Visceral fat distribution
WHtR	Central adiposity relative to height
Conicity index	Body shape / visceral fat loading
AVI	Abdominal volume / geometry
BRI	Abdominal roundness / geometry
LAP	Visceral fat–related metabolic load
VAI	Visceral adiposity + lipid profile
TyG index	Insulin resistance / lipid–glucose dysregulation

Abbreviations: BMI, body mass index; WHtR, waist-to-height ratio; WHR, waist–hip ratio; AVI, abdominal volume index; BRI, body roundness index; LAP, lipid accumulation product; VAI, visceral adiposity index and TyG index, triglyceride glucose index.

Supplementary Table S2. Variance Inflation Factors (VIFs) for Obesity-Related Indices in Sex-Stratified Logistic Regression Models

**Obesity-Related Indices**	**Male**	**Female**
	**VIF***	**VIF***
BMI (kg/m2)	4.703	3.309
WC (cm)	1.161	1.164
WHtR	1.186	1.215
WHR	1.216	1.208
AVI	1.158	1.157
BRI	1.180	1.207
LAP	1.115	1.153
VAI	1.053	1.081
Conicity index	1.156	1.131
TyG index	1.150	1.250

Abbreviations: VIF, variance inflation factors; BMI, body mass index; WHtR, waist-to-height ratio; WHR, waist–hip ratio; AVI, abdominal volume index; BRI, body roundness index; LAP, lipid accumulation product; VAI, visceral adiposity index and TyG index, triglyceride glucose index.

* VIF values were calculated from models including one obesity-related index at a time along with covariates. A VIF < 5 indicates no concerning multicollinearity.

Supplementary Table S3. Hosmer-Lemeshow Goodness-of-Fit Statistics for Sex-Stratified Logistic Regression Models

**Obesity-Related Index**	**Male χ^2**	**Male p-value**	**Female χ^2**	**Female p-value**
BMI (kg/m^2)	16.375	0.037	22.730	0.004
WC (cm)	12.380	0.135	18.911	0.015
WHtR	6.580	0.583	18.485	0.018
WHR	2.512	0.961	26.068	0.001
AVI	12.166	0.144	21.282	0.006
BRI	7.180	0.517	19.775	0.011
LAP	7.982	0.435	25.432	0.001
VAI	12.049	0.149	18.629	0.017
Conicity index	5.352	0.719	22.492	0.004
TyG index	6.083	0.638	15.969	0.043

Abbreviations: VIF, variance inflation factors; BMI, body mass index; WHtR, waist-to-height ratio; WHR, waist–hip ratio; AVI, abdominal volume index; BRI, body roundness index; LAP, lipid accumulation product; VAI, visceral adiposity index and TyG index, triglyceride glucose index.

Supplementary Table S4. Comparison of adjusted odds ratios (ORs) and 95% confidence intervals for psychiatric morbidity across ten obesity-related indices, by sex.

Index	Men OR (95% CI)	Women OR (95% CI)
BMI	1.05 [0.98, 1.12]	1.03 [0.97, 1.10]
WC	1.18 [1.10, 1.27]	1.10 [1.03, 1.18]
WHtR	1.42 [1.32, 1.54]	1.28 [1.19, 1.38]
WHR	1.45 [1.34, 1.57]	1.26 [1.16, 1.36]
AVI	1.22 [1.13, 1.32]	1.15 [1.06, 1.25]
BRI	1.25 [1.16, 1.36]	1.17 [1.08, 1.27]
LAP	1.28 [1.19, 1.38]	1.21 [1.12, 1.31]
VAI	1.14 [1.06, 1.24]	1.09 [1.01, 1.17]
Conicity Index	1.55 [1.43, 1.68]	1.31 [1.20, 1.42]
TyG Index	1.21 [1.13, 1.30]	1.14 [1.06, 1.23]

Abbreviations: VIF, variance inflation factors; BMI, body mass index; WHtR, waist-to-height ratio; WHR, waist–hip ratio; AVI, abdominal volume index; BRI, body roundness index; LAP, lipid accumulation product; VAI, visceral adiposity index and TyG index, triglyceride glucose index.
